# Collectanea Medica, Consisting of Anecdotes, Facts, Extracts, Illustrations, Queries, Suggestions, &c.

**Published:** 1815-05

**Authors:** 


### COLLECTANEA MEDICA,

CONSISTING OF

### ANECDOTES, FACTS, EXTRACTS, ILLUSTRATIONS, QUERIES, SUGGESTIONS, &c.

RELATING TO THE

*History or the Art of Medicine, and the Auxiliary Sciences.*

THE following Account of Egypt is taken from Baron Grimm's celebrated Memoirs. We have thought it right to copy it, not merely on account of the curious facts and well-founded conjectures with which it abounds, all of which relate to the health of the place, but on account of one epidemic disease which is now constant in that country, and of which, in its more flourishing days, history gives us no information. Is it likely that the true plague could have been unknown at those times? Is it not more probable, that, during the plague season, all the initiated, who consisted of all the best informed part of the population, retired to these these retreats, which secured them from the concentrated effects of a pestilential atmosphere, by inducing a constant current from the different temperature within and without? From the caution given to Herodotus, we may conjecture that only certain individuals, secured, by the priests, by charms or incantations, could venture into these parts. Even the fable of the sphynx, who devoured all that did not comprehend her mysteries, was, probably, only an anathema against such as ventured into the labyrinth without proper instructions. Should Egypt again become the seat of our arms; above all, should that most central of all spots on the habitable globe become a British settlement, would it not be desireable to explore these curious retreats, and see how far art can supersede the only alloy attendant on so highly favoured a region?

M. Garat's *Account of the Pyramids of Egypt.*

The most favoured climate in the world has its inconveniences: even Egypt is not without its evils, notwithstanding its great fertility. Though its situation only approaches the tropic, it endures more heat than the torrid zone in many parts of the globe. Those frequent rains and seasonable storms which temperate and refresh the heated air of the tropics, are here almost unknown. A cloud is never to be seen between the sun and the earth; and the rays of the sun, concentrated and reflected, fall perpendicularly on the two chains of mountains which follow the course of the Nile, becoming like a burning mirror, which spreads its flames far and wide; for, whilst the heat pursues you, the land offers no retreat.

Egypt is entirely without high trees: it boasts none of those forests whose waving acts as a ventilator in the torrid zones, and whose elevated and umbrageous summits arrest the sun's rays, and keep up a steady coolness below, during the intense heat that reigns above. The earth, impregnated to a great depth by the waters of the Nile, is rendered fertile by this great heat; but the living creatures suffer continually from it. There are parts of the year when the animals who feed in the neighbouring plains are parched as by a fire, fill the air with their cries, and rush into the waters of the Nile, where they remain night and day. The buffalo, the hog, the horse, and the ox, are there almost amphibious; it would appear that Egypt, at certain periods, had no other living creatures except fish. It was in Egypt that a Frenchman wrote the singular work called *Tellamied,* where he pretends that all the animals, and even man, commenced their origin as fish. The men and women certainly live in the waters of the Nile very much with the fish. One is surprised to see the number of children and young people swimming across and amusing themselves about this river and its canals. In the time of Herodotus and Thales, they were seen rising from the water, and surrounding the boats which came up and down the Nile, accompanying them with their songs. This sight, one would suppose, gave rise to the fable of the Nereids.

Homer

Homer saw it: Homer's genius was formed in part by what the nature of Egypt offered to him.

But this climate has something still more terrible than its burning heat, a scourge that the waters of the Nile cannot prevent, and which deprives the inhabitants from seeking refuge in the river. I mean those violent winds which come from the sandy deserts of Arabia, by which Egypt is surrounded. In one moment the ground and all the atmosphere is filled with a sand which appears to have been reddened by fire, and which penetrates the smallest interstices of the walls and other enclosures. The houses do not even afford shelter, and often whole families have been buried in their beds by these torrents of burning sand. There is no security for the inhabitants at those times but the earth, which the Egyptians and Africans in general resort to for safety. These people have always lived a good deal under ground, and those subterraneous and gloomy dwellings which frighten our imaginations, are to them most delightful asylums. In almost the whole extent of Africa, the climate at times has rendered these habitations necessary and agreeable. When Hanno left Carthage on his voyage of discovery like the modern Cook, he saw, at night, fires all along the western coast of Africa, and heard the sound of musical instruments, of dancing, and singing. As soon as the sun appeared in the heavens, all was silent; no human being was heard or seen; all appeared a desert, abandoned to the sands and the influx of the sea. All the people at that time were taking refuge in their caverns. At the other extremity, on the eastern coast, we have seen the Ethiopian Ichthyophagi only roused from their stupid indolence to search for caverns which the sun could not penetrate; we have seen them construct artificial rocks with sea-weed and sand, resembling a rough pyramid. In Upper Ethiopia, and near the cataracts, the ground is covered by deep excavations made for the habitations of the natives. It was here the Ethiopian priests made their sacrifices and their initiations; and here some of them passed their lives without ever seeing the heavens, that sun and those stars which they adored. The Ethiopians, as descendants of the Egyptians, preserved their taste for these dwellings, which became more necessary to them among the calcined rocks of Lybia and Arabia. Thebes, with its hundred gates, was at first a subterraneous city: its first street and dwellings were formed out of two parallel rocks to the right and left of this capitol. Those which were called the king's burying places, were, in fact, a subterraneous country, where a whole nation could be accommodated, and where were to be found innumerable galleries, halls, palaces, and temples. These caverns might also have been used as royal burying places; but I must place some confidence in history, which says expressly, that the first Theban kings lived there. We may also admit, that the same building afterwards accommodated both the living and the dead.

A number of temples in Egypt were excavations from rocks. (See in Diodorus Siculus a long account of the tomb of Osimander.)

 That

That description gives us a true idea on the subject, which the word tomb hides from us. There are still to be seen in Egypt several other edifices, by different names, resembling Osimander's tomb; such as the Labyrinth, the most celebrated of all the famous Egyptian edifices which Herodotus speaks of as having seen and visited. This place served as a rendezvous for the kings, when there were twelve in Egypt, as well as for the priests, and the nation in general, to deliberate in. It appears that the Labyrinth contained the same number of apartments under ground as above, which were innumerable. Herodotus wished to explore them, but was prevented by his guides, being told there were in these caverns crocodiles, consecrated to the sepulchres of those kings who had constructed the labyrinth.

From all these circumstances, M. Garat concludes, that these immense dwellings were intended principally to protect the priests and the people in their public ceremonies, whether political or religious, from the intense heat of the sun, and the whirlwinds of burning sands, which penetrated to the interior of all their other buildings. More than half, adds he, of these pyramids was under ground; and even that part which was raised to six hundred feet above ground, formed of enormous rocks thirty or forty feet thick, closed all round hermetically, was only as it were a sort of cavern raised above ground. There are still some breathing places to be found, which were intended, no doubt, to renew the air of the pyramid at those times when the external air was the coolest. It was here the Egyptian priests retired to meditate on their gods, and to make new ones, and to take measures against the usurpations of some of their kings, as well as to celebrate those famous mysteries of antiquity. These dark abodes were well calculated to inspire the enthusiastic mind with terror. Edifices raised so high in the air, and so low under ground, were admirably contrived to persuade the initiated that he was at times raised to the heavens, and at others precipitated to the infernal regions. Long tunnels and galleries, where the report of a pistol-shot is reverberated in continued echoes, like the noise of a cannon, were well constructed to make the initiated fancy he heard the sound of thunder. In a word, I am persuaded that these buildings served for many purposes of society, as well as all the other buildings of the same kind.

There were two Egypts, one above, the other under, ground; and the pyramids partook of each. Whether above or below, they always served to protect the natives from the too great scourges of their climate, the parching heat of the sun, and the gusts of burning sand. This conclusion may not be approved, but I have drawn it from the nature of the climate, from the general plan of the Egyptian architecture, from their taste for subterraneous dwellings, from their religious ceremonies, and from all that history relates of the prodigies of their initiation.

---

That the vast difference in the seasons produces the necessity for such such retirement, is sufficiently proved by the very accurate description collected from Dr. Clarke.* "Villages," says that interesting writer, "in an uninterrupted succession, denoted a much greater population than we had imagined the country contained. Upon each side of the river, as far as the eye could survey, were rich corn and rice, with beautiful groves, seeming to rise out of the watery plains, and to shade innumerable settlements in the Delta, amidst never-ending plantations of melons and all kinds of garden vegetables.

"Upon the retiring of the Nile," continues he, "this country is one vast swamp. An atmosphere impregnated with every putrid and offensive exhalation, stagnates, like the filthy pools over which it broods. Then the plague regularly begins, nor ceases until the waters return again. Throughout the spring, intermittent fevers universally prevail. About the beginning of May, certain winds cover even the sands of the desert with the most disgusting vermin. The latest descendants of Pharaoh are not yet delivered from the evils which fell upon the land, when it was smitten by the hand of Moses and Aaron; and the plague of frogs, the plague of lice, the plague of flies, the murrain boils and blains, prevail so, that the whole country is corrupted, and the dust of the earth becomes lice upon man and upon beast throughout the land of Egypt." To prove this, it is only necessary to visit the country at different seasons. During the months of June, July, and August, there was hardly an individual who did not suffer, more or less, by the ophthalmia.

In the same chapter he remarks, "The mercury remained fixed at 90° for several days, without the smallest perceptible change. Almost every European suffered an inflammation of the eyes. Many were troubled with cutaneous disorders. Many persons were afflicted with boils on their skin, which were called boils of the Nile; and dysenteric complaints were universal. The plague of flies covered all things. It was impossible to eat without hiring persons with flappers at every table. Liquor could not be poured into a glass: the mode of drinking was by keeping the mouth of every bottle covered till the moment it was applied to the lips, and removing it to another covered with the palm of the hand. No cleanliness could secure you from vermin. A gentleman made his appearance before a party he had invited to dinner, covered with lice; the only explanation he could give, was, that he had sat for a short time in one of the boats upon the canal."

After this account, can we wonder if, in such a country, the whole business of life was to find a retreat from the external atmosphere during the summer season. In the winter, the temperature is suited to the plague; but from this there is a resource in well-ventilated apartments. In summer, ventilation is only the admission of a noxious air and disgusting insects. At a period, therefore, when labour was of little value, and almost the whole

* Travels in Africa, &c. sect. 2, part 2, chap. 2.

population might be directed to a single object, it is at least less wonderful that such erections should be raised. It may, perhaps, be asked, why they are not still appropriated to the supposed original purposes. The answer to this is not very difficult. From the time that we have distinct records of Egypt, it has been a conquered country, and maintained in subjection by an inconsiderable armed force, which has been engaged only in oppressing the natives, and preventing iusurrections. During centuries of this unnatural state of existence, no wonder if the habits of the native princes, with those about them, have been lost, whilst, probably, the servile race, and even the plebeian class, may have gradually lost every idea of luxurious refinements, by the impossibility of acquiring them. Should Egypt ever again be governed by wholesome and equal laws, can we doubt that the wealthy will discover some means of avoiding the constant annoyance above described. Can we conceive any retreat better calculated for a long and uninterrupted serenity of sky, suited only to those insects which bask in the sun-shine, and whose unrestrained multiplication must be principally supported in their successive generations by feeding on the larger animals.

---

*Account of the Proceedings of the French Institute in the Departments of Physiology, Anatomy, and Surgery, for the Year* 1814. By M. Cuvier.

M. Dutrochet, a physician at Chateau Renaud, has generalized his researches and presented the results to the Institute, in a Memoir on the Envelopes of the Fœtus, of which we shall now give a sketch, subject to the remark, that the committee of the Institute have not yet been able to verify these results.

The author thinks he has discovered, that, in the earlier stages, the fœtus contained in the egg has an aperture at its abdominal parietes, and at its amnios, through which there passes an extension of the bladder, which forms the chorion and the intermediate membrane: so that the umbilical vessels are nothing but productions of the vessels of the bladder. According to M. Dutrochet, the egg of the reptile genus is a vitellus deprived of albumen; and, in the viper, the membrane of the shell, which is extremely thin, disappears towards the middle period of gestation, the bare chorion then contracts adhesion with the oviductus, without forming, however, a true placenta. Thus this membrane of the shell would be analogous to the *deciduous membrane* of the mammiferæ. He asserts, that the bull-headed frog does not throw off its skin to metamorphose itself, but that the anterior paws pierce this skin, that the teeth tear it, and the apertures cicatrize. The egg of the frog is a vitellus, the emulsive matter of which is contained in the intestine itself, which, at first globular, lengthens by degrees into a spiral tube, as we see it in the bull-headed frog. M. Dutrochet has also some peculiar ideas as to the respiration of the fœtus, and particularly as to the gills of the frog, which he thinks thinks are placed within the cavity of the tympanum. We shall speak of his paper more at length, when the season arrives to admit of our repeating his observations from nature.

Comparative anatomy had never determined in a positive manner the nature of the respiratory organs of the cloportæ. We know, that these animals resemble greatly in structure the crustacea ; there was reason to suppose, that the laminæ placed under the tail served for respiration, as they certainly do in craw-fish, animals which resemble the cloportæ much. But it remained to ascertain the fact, and to shew at the surface or in the inside of their bodies any apparatus for performing this function.

M. Latreille, who has been recently elected into the Institute, has filled up this hiatus in zoology. He has shewn on four of the laminæ in question a small yellowish part, pierced with a hole, and containing in the inside small filaments, a part which he compares to those, which although differently placed in the spider and scorpion, have nevertheless an analogous structure and answer the same purpose. Notwithstanding this partial resemblance and the existence of a kind of web apparatus, which he observed in the cloportæ, and which is an additional connection with the spider, M. Latreille does not separate the cloportæ from the crustacea, on account of their other much more numerous analogies to this class.

Insects have been long divided into two categories, according to the structure of their mouths, some having teeth well developed and which can divide solid food, while others have trunks or suckers adapted for liquids only. There are even some which, at different periods of their lives, assume both these forms of mouth, and in which a metamorphosis changes into suckers, when in their perfect state, what were masticaters in their state of larvæ: such, for instance, are the butterflies, which feed by a double trunk only, generally spiral, which they unroll in order to introduce it into the bottom of the corollæ of flowers to suck the nectar ; whereas caterpillars, which are butterflies not yet developed, have their mouths armed with strong teeth, with which they cut the hardest leaves. Thus it has been supposed, that the caterpillar in taking the wings, the long legs, and the beautiful antennæ of the butterfly, also took its trunk and lost entirely its teeth.

M. Savigny, the member of the Institute of Egypt, has proved by some delicate and accurate observations, that this is not the case entirely, but that nature in this as well as in many other cases, confines herself to altering certain parts in order to develop others from them ; thus producing effects entirely opposite by these simple changes of proportion. He has discovered, at the basis of the trunks of the butterfly, two organs of extreme minuteness, but which nevertheless represent the teeth of the caterpillar ; at the back of the support of this same trunk, he found two very small filaments, which appeared to him to be analogous to the maxillary palpæ, so that the two laminæ of which the trunk is composed, are, according to M. Savigny, the points of the the maxillæ extremely elongated, i.e. of the lower pair of grinders. Finally, the great feelers known to all naturalists are the palpæ of the lower lip. The two small maxillary feelers had been already perceived in some kinds of night butterflies; but we are indebted to M. Savigny for the information that they exist in the whole family. This acute observer has also established a marked analogy between the bristles and some other minute parts, which generally accompany the sucker of insects with two wings, and the mandibles and the maxillæ of the masticating insects: so that the structure of this numerous class of animals presents in this important part of their organization, a more satisfactory uniformity than has been hitherto supposed.

M. Savigny also examined the mouths of insects, which, in addition to palpable grinders, have a trunk formed by the prolongation of their lower lip, insects of which the bees are the most remarkable. It had been supposed, that the aperture of the pharynx was situated under the trunk or lip, whereas, in the common masticating insects, it was above; but this is a mistake: the pharynx is always on the basis of the trunk, and it is even there furnished with parts interesting to be acquainted with, and of which M. Savigny gives a detailed description. His memoir is intended for the great work upon Egypt which is about to be terminated in consequence of the *munificence of the king*.

M. Cuvier has made some researches concerning another class, the mouths of which also present, in appearance at least, numerous anomalies, viz. fishes. Here we find every part which belongs to quadrupeds; but some are more subdivided, and their subdivisions are sometimes reduced to such a minuteness that they cannot perform their functions, and we have even a difficulty in perceiving them. The greater number of fishes have intermaxillaries and maxillaries which are very visible; but these bones differ much from each other in point of proportion; and the maxillaries in particular sometimes form part of the edge of the jaw, and have teeth, and sometimes they are placed farther back and have no teeth, a circumstance from which naturalists have erroneously named them mystaceous, or labial bones. These differences afford the author generic characters very convenient for effecting a more natural distribution of the species, but they cannot serve for distinguishing the orders. For this latter purpose, M. Cuvier has recourse to more striking differences, such as the coalition or adhesion of the maxillaries with the intermaxillaries, which takes place for instance in the tetradons, the coffri, and the balistæ, or such as the separation of the one from the other, and the obligation upon nature to employ the palatal bones in forming the upper jaw, which is observed in the raii, the squale, &c.

The author was not able to discover any other characters than those for establishing a first distribution of the class of fishes. In consequence, he refers to the common fishes the genera, which, having the same structure, mouth, and gills, had, nevertheless, been been placed among the branchioslegal or cartilaginous fishes, on account of some singularities of external form, or because their skeleton took a little more time to harden than the others; such are the centrisci, the *cyclopteri*, the lepagadasteri, &c.

M. Cuvier has founded upon these and similar views, the peculiar methods according to which the fishes will be distributed in the work which he is preparing upon comparative anatomy.

The same ingenious naturalist has also presented to the Institute Observations on a great number of Species of Fishes, collected during Three Voyages along the Shores of the Mediterranean. Some are new, others had been erroneously named or distributed by various authors; many of them presented a curious structure, or gave rise to the establishment of new genera, or to the subdivision of the old genera. These details cannot enter into our present report, but naturalists will find them in the first volume of the Memoirs of the Museum of Natural History, of which one number has already appeared.

M. Risso, the author of the Ichthyology of Nice, has transmitted to the Institute a supplement to that work, in which he describes several fishes with which he was not before acquainted, and which are very curious and interesting.

M. Lamouroux has extended and brought to perfection his great work on the Polypi, which we mentioned two years since, and which will speedily be published.

All must recollect the fine experiments of M. Majendie upon vomiting and the invitation given him by the class to examine what part was acted by the œsophagus in this disordered movement of the stomach. Although his researches have not yet afforded him any decisive results, they appeared to him to be sufficiently interesting to communicate.

The alternate constrictions and relaxations of the œsophagus appeared to him to take place in the inferior third part only, where it is chiefly animated by the nerves of the eighth pair. The constriction increases much and lasts long when the stomach is full. When the œsophagus is cut and detached from the diaphragm, the injection of an emetic into the veins produces no more vomiting, and its immediate introduction into the stomach becomes necessary for this purpose.

M. Delpech, the Professor of Surgery at Montpelier, has sent to the Institute a Memoir on the Putrescency of Hospitals, a species of gangrene which attacks wounds when the patients are too much crowded together. He is convinced that this formidable symptom, which few practitioners have mentioned, essentially proceeds from a local contagion, and is propagated by the linen, the dressings, and the instruments. Its progress is checked when the wounded are removed or exposed to a current of air: the utmost attention to cleanliness are necessary to prevent its spreading; but the only true remedy, according to M. Delpech, is to destroy life, by the actual cautery in the parts which are affected by it.

Some years ago, M. Maunoir, a Surgeon of Geneva, sent in a Memoir

Memoir on the Advantages of the Method of Amputation invented in England, and which consists in cutting the skin much lower than the bone and muscles, and in such a way as to preserve enough to cover the stump by bringing all close together immediately after the operation.

M. Roux, a Surgeon of Paris, has presented a Paper on the same subject, in which he shews, from his own experience, that this method diminishes the sufferings of the patient, that it prevents hæmorrhage and suppuration, accelerates the cure of the wound, and leaves the stump in a more convenient state and less liable to accidents. He points out the precautions necessary to avoid some inconveniences which occur to those who operate badly, and particularly that of leaving a sufficient issue for the blood and pus. M. Percy, our associate, who has practised this operation since his youth, and who, as he informs us himself, has had the sad advantage of performing more amputations, perhaps, than any surgeon who ever lived, expresses, in his report, an ardent wish that M. Roux should make public his valuable improvements.

Two young surgeons of Paris, Messrs. Lisfrand and Champesne, have made known a method which they have contrived for the amputation of the arm in its upper articulation, one of the most difficult operations in surgery. They insert the instrument under the two pre-eminences of the omoplata called the acromion, and the coracoid apophysis; they cut immediately into the articular capsule, and finish the operation quicker than by any of the processes hitherto employed.

M. de Saissy, a Surgeon of Lyons, has obtained successful results in several cases of deafness by throwing injections into the cavity of the tympanum through the eustachian tube: he has transmitted a description of his method and a history of the cures which he has effected.

M. Orfila's Treatise on Poisons, the first volume of which we mentioned last year, has been continued, and the second volume submitted to the class in manuscript. It treats of the deleterious effects of preparations of tin, zinc, silver, and gold, as well as of the concentrated mineral acids, the caustic alkalies, phosphorus, cantharides, lead, and iodine; and contains an appendix on the counterpoises of corrosive sublimate and arsenic. The author herein carefully details, according to new and accurate experiments, the physiological effects of these substances, when swallowed or injected into the veins.

Milk, according to M. Orfila, is the antidote to the muriate of tin; sea salt to the nitrate of silver, or lapis infernalis; calcined magnesia to the acids, if it be employed instantly; the sulphate of soda and magnesia, or Epsom and Glauber Salts, when taken in large quantities and repeated doses, stop the effects of the salts of lead and barytes, and the acetic acid is the best remedy against the action of the alkalies.

M. Orfila proves that charcoal, which had been recommended against against corrosive sublimate and arsenic, can be of no use: it is gaining much to be made acquainted with the inefficaciousness of a remedy against mischief, when we have no time to try fruitless experiments.

M. Huzard has carefully informed the Institute of the progress and termination of a dreadful disease which carried off almost all the horned cattle in the provinces of France which were subjected to the horrors of war. It was a bilious and putrid fever, which, without coming from Hungary, nevertheless appeared wherever the bullocks of that country were driven in the rear of the armies. The strict interruption of all communication was the only efficacious preservative, but no remedy could save the poor animals which were attacked; happily, their flesh was wholesome, which diminished a little the loss of the proprietors. The same gentleman also read a notice of a disease which appeared among the cattle of the village of Rosny, and which had been taken for hydrophobia; he ascertained, however, that it was only a gangrenous sore throat.

It may seem invidious to make any remarks on the above memoir. On the physics, or as we usually term it natural history, we have nothing to object; but, whilst we acknowledge, that the French were somewhat more than a century ago our masters in surgery, and that their dexterity may still give them some advantage in manipulation, can we fail to express our astonishment at this mention of the hospital gangrene and flap operation, each of them accurately noticed in Britain more than twenty years past?

---

We cannot fail to express our disgust at the cruel manner in which the French physiologists continue their unnecessary experiments on living animals, merely to prove what was considered by the late Mr. Hunter, if not self-evident, at least proveable, by accurately tracing the actions as they occur in the various operations produced. To shew this we shall copy a passage from his last edition of Animal Economy.

"It may be remarked, that the motion of the whole intestinal canal, from the fauces to the anus, is naturally so slow, as not to be excited into quick actions. The food passes slowly along the œsophagus; and in a man, fluids which might be expected to act even by their own gravity, descend but slowly: yet I think we may be certain that the œsophagus has always a regular contraction; and that the lower parts must relax in progression, as it contracts above; so that no position of the body makes any difference in this action.

"Upon exposing the stomach in living animals, it does not appear much agitated or affected, even by being handled or otherwise irritated. The same thing may be observed in the whole track of intestines: and we find that when the fæces are expelled by the action of the gut alone, that the expulsion is slow; the stomach and rectum, however, can be emptied at once; but that is done by the the abdominal and other muscles. We know that the action of vomiting is performed entirely by the diaphragm and abdominal muscles; and we know that by the same action the contents of the rectum can be expelled. Neither is any other power required to empty the stomach in vomiting, these muscles being often capable of forcing the bowels themselves out of the abdomen, and of producing a rupture. It is not necessary the stomach itself should act violently to produce an evacuation of its contents; nor is it even necessary it should act at all: for the lungs themselves do not act in the least when any extraneous matter is to be thrown up; and coughing is to the lungs, what vomiting is to the stomach. The muscles of respiration are the active parts in emptying the lungs, and can act both naturally and preternaturally. The muscles of the thorax and abdomen do not act naturally on the contents of the abdomen, but often act preternaturally, producing an evacuation from its viscera.

"There is this difference in the action of the parts in coughing and vomiting: the cough is performed by the proper muscles of respiration, which are those of expansion, supported by the abdominal, while the diaphragm is passive.

"Vomiting is performed by the abdominal muscles and diaphragm; while those of inspiration are supporting this action.

"In coughing, the ribs are suddenly depressed, which diminish the capacity of the thorax; and that the diaphragm may not be allowed to sink down and increase the capacity of the thorax, which would counteract the depressors of the ribs; the abdominal muscles at the same time act, which supports the diaphragm in its place, and probably may by this action assist in bringing down the ribs. To give as much force to this action as possible, the glottis is shut till the action is begun, and then the glottis opens instantaneously, which obliges the depressors of the ribs to begin the effort with their full action.

"The proper muscles of inspiration do not tire so soon in this action as the abdominal; for in violent coughing the muscles of the abdomen become sore.

"In vomiting, these actions are reversed. The muscles of the cavity of the abdomen act, in which is to be included the diaphragm; so that the capacity of the abdomen is lessened, and the action of the diaphragm rather raises the ribs; and there is also an attempt to raise them by their proper muscles, to make a kind of vacuum in the thorax, that the œsophagus may be rather opened than shut, while the glottis is shut so as to let no air enter the lungs. The muscles of the throat and fauces act to dilate the fauces, which is easily felt by the hand, making there a vacuum, or what is commonly called a suction; so that, when all these actions take place together, the stomach is immediately emptied.

"In violent coughing, we find that a kind of mixed action takes place; for, although the diaphragm has not acted, yet the stomach is so much squeezed as to discharge its contents; and it affects the diaphragm, which is often thrown into action, and brings on vomiting at the same time; therefore violent coughing palls the stomach."*

The only apology we can make for the French physiologists is, that the above paper is not contained in the first edition of Mr. Hunter's Observations on certain Parts of the Animal Economy, nor in his paper in the Philosophical Transactions. Probably the last may be all that they have seen of his opinions on digestion.

A well arranged edition of all Mr. Hunter's medical works is much wanted.

---

*On the Treatment of Fungus Cerebri;* by JOHN and CHARLES WENZEL.—*Translated from Journal de Med.*

To enumerate the external means of treating these affections is to take a very wide range in the field of surgical application. The taxis, an altered position of the head, bandages, fomentations, plasters, caustics applied to the integuments, incision, puncture, ligature and excision of the tumour, trepan, scarification, exsiccating, catheretic powders, and the actual cautery, have severally been recommended; in short, scarcely any thing has been omitted.

The mere handling of these tumours discovers to us that it is in general easy to return them into the cranium; but stupor, fainting, and convulsions, are often the consequence. When the roughness of the osseous ring surrounding the fungus produces irritation in it, the reduction may lessen the pain, and may often be promoted by a change of position; and, by means of bandages, the tumour has been confined within the skull, but it is a precarious procedure, attended with many inconveniences, and of very doubtful efficacy in any case.

An empiricism still more gross, established the use of different emollient, resolving, and irritant applications, each of which have had their trial. Ignorance has even gone the length of expecting advantages from methods of revulsion. Plasters have been applied to the soles of the feet, and blisters to the knees.

The employment of caustics to open these tumours, owes its birth to the erroneous opinion of their being cysts, whose abscission ought not to be attempted. This rash manœuvre has been followed by a hæmorrhagy that could not be restrained,† or by mortal convulsions.‡ To plunge a cutting instrument into their substances, was a resolution no less adventurous. This operation has more than once been fatal. In some, death has followed immediately;§ in others, at a period somewhat later,‖ even after favourable symp-

---

* Animal Economy, p. 199.

† Kauffman, Dissert. de Tumore capitis Fungoso, post cariem cranii exorto. Helmstadii, 1743. Vid. Haller, Disp. Chirurg. Select. tom. i. p. 49.

‡ Legrand, Memoirs of French Academy of Surgery.

§ Ambrose Paré, Siebart, Chopart. Id.

‖ Rey and Philippe. Id.

toms were discovered. The symptoms which terminated in death were hæmorrhagy and convulsions, as in those treated by caustic.

This method, often pernicious, always inefficacious, when confided in *alone*, was much improved by the practice of applying the trepan in several parts of the circumference of the tumour, that it might be enveloped in its whole content. Marc. Aurelius Severinus* advises the removal of the bony parts which cover the roots of the disease, after which the remedies are to be applied. Marregues† also proposed the same practice a little time afterwards. Volprecht, in one case, confined himself to the operation, as it was not agreed what else should be done to the fungus;‡ and,§ by an incision through the scalp, discovered both the fungus and the foramen, through which it escaped: then he removed, by the trepan and cutting pincers, the portions of skull which confined the base of the disease, and succeeded in effecting the cure. Siebold‖ gives the same advice, and performed the operation himself; but its success was obstructed by the previous handling of the tumour, which burst, and occasioned profuse hæmorrhagies. The patient died in violent convulsions. The abscission of a part of the tumour, leaving the remainder to be destroyed by suppuration, cannot be recommended, having frequently occasioned inflammation of the subjacent membrane of the brain. Salzman¶ has seen this operation followed, in eleven days, by bilious vomitings, delirium, and death. Constant experience of the failure of a simple incision in the substance of these tumours, led to the attempt at complete eradication. For this purpose have been employed the knife and ligature, catharetics and caustic powders, and even the actual cautery. The removal by the knife was considered applicable to fungi of small size, encased, as Louis observed, in a duplicature of the dura mater.** In this case, as in those of Marregues and Volprecht, the operation, according to Louis, should consist in a simple incision of the enveloping membrane, and in the total recision of the fungus, without touching the internal lamina of the dura mater. Others have proposed to remove the portion of membrane covered by the disease, to prevent a return. If the excrescence is large, Louis advised, from Ambrose Paré,†† a ligature to be applied to its base. Pohl‡‡ has given the history of

---

* De Medecina efficac. lib. i. part 2, cap. 3. Chirurgia quæ ad ossa pertinet.

† Memoirs of French Academy.

‡ Ibid.

§ Stolz, præside Sand, de fungo cerebri Regiomonti, 1700. Vid. Haller, Disputation. Chirurg. Selec. Tom. prim. Amstelodami, 1755. P. 172.

‖ Arneman's Magazin. vol. i. p. 402.

¶ Mem. French Acad.

** Ibid.

†† On particular Wounds, chap. xxi.

‡‡ Acta. Erud. Lips. ann. 1736, mens. maii.

a case a case where the excision was not deemed adviseable, on account of an evident pulsation in it. The ligature was therefore employed. It was tightened daily. In fifteen days, the base of the tumour, which was thirteen inches in circumference, was reduced to one. Notwithstanding, the patient died in convulsions. The effect of escharotic remedies applied to excrescences which arise from injuries of the dura mater or cranium, has led to their employment in the real fungus cerebri, either after making the incision into the enveloping membrane, or later, when the knife or ligature has removed the greater part of it. It is the latter case, where the substances are not too active, in which they are most likely to be serviceable. Caustics employed in the same circumstances have always produced bad effects—fever, inflammation, violent pain, delirium, convulsions, and death. The actual cautery, still more powerful, is also more to be dreaded, on account of its action on the neighbouring organs, how efficacious soever it may be against existing hæmorrhages. Their employment is contra-indicated by the production of symptoms still more dangerous.

*Proposed method of cure.*—When the disease has become evident to the sight and touch, the operation is the only means on which we can depend, and especially when symptoms are present that threaten the life of the patient. The reduction can never be thought of unless when the tumour is irritated by the margin of the surrounding bone, nor should we persevere in this if the symptoms are increased by it.

In performing the operation, our incision should be made in the integuments sufficiently free to lay bare the whole of the diseased bone. When the disease is exposed to view, we discover the state of the margin of the bone, and the relative size of the tumour with the aperture. Often we see also the direction which the excrescence takes beneath the bone that covers it.

If the perforation of the bone is smaller than the base of the fungus, we must enlarge the opening by means of the trepan. If, on the contrary, we are enabled freely to discover the whole extent of the disease, we should proceed immediately to its extirpation. Experience teaches that in many cases this is easily effected by the want of adherence of the excrescence to the membrane, that it requires no effort; and in many cases, after the removal of the disease, the membrane is found not to be diseased. Where it is necessary to have recourse to excision, it must be done with great care not to injure the continuous parts. Caustics and the ligature are highly to be reprobated, for the reasons before-mentioned.

If the fungus does not merely adhere to the dura mater, but is included in its laminæ, a portion of this membrane must be removed as Louis advises. The small portions which cannot be removed without violent means, should be left to the suppurative action, which procures their dissolution.

The best mode of repressing hæmorrhagy, whether this arises from the peculiar structure of the fungus, or from the diseased condition of the place which gives rise to it, is to terminate the operation operation as speedily as possible. Should the tumour adhere to the inequalities of the margin of the bone, it should be detached as carefully as before.

It is deserving of consideration, whether the trepan is adviseable in cases where all the symptoms indicate the existence of a fungus. Although the bone, diseased, swollen, and carious, presents no opening, this frequently happens in fungi, arising from an internal cause.

The diseases liable to be mistaken for fungus cerebri, are exostoses and periostoses of the cranium, horny excrescences, encysted tumours, cerebral herniæ, diseased state of the vessels of the dura mater, osseous concretions, and scrofulous tumours of the dura mater.

### CRITICAL ANALYSIS

### OF RECENT PUBLICATIONS

IN THE

